# Evaluation of Thimerosal Removal on Immunogenicity of Aluminum Salts Adjuvanted Recombinant Hepatitis B Vaccine

**Published:** 2012

**Authors:** Arash Mahboubi, Mohammad Reza Fazeli, Nasrin Samadi, Rasoul Dinarvand, Saeed Azadi

**Affiliations:** a *Department of Pharmaceutics, School of Pharmacy, Shahid Beheshti University of Medical Sciences, Tehran, Iran.*; b *Department of Drug and Food Control, Faculty of Pharmacy, Tehran University of Medical Sciences.*; c *Department of Pharmaceutics, Faculty of Pharmacy, Tehran University of Medical Sciences.*; d *Drarou Pakhsh Pharmaceutical Mfg. Co. Biotech. Research Department Tehran, Iran. *

**Keywords:** Hepatitis B vaccine, Thimerosal, Aluminum salts, GMT, Relative potency

## Abstract

Thimerosal, which is approximately 50% mercury by weight is a preservative widely used in vaccines since the 1930’s. It meets the requirements for a preservative as set forth by Pharmacopeia challenge test and has been shown to be effective against a broad spectrum of pathogens. In July 1999, the Public Health Service agencies and vaccine manufacturers agreed that thimerosal should be reduced or eliminated in vaccines as a precautionary measure but, due to the lack of appropriate alternative, it is still extensively used in multiple dose formulations of vaccines such as hepatitis-B in developing countries. In this study the effect of the removal of thimerosal in two formulations of hepatitis B vaccines containing either aluminum hydroxide or aluminum phosphate were evaluated in Balb/c mice. These formulations were administered interperitoneally and the titer of antibody was determined by ELISA technique after 28 days.

The geometric mean of antibody titer (GMT), seroconversion and seroprotection rates, ED50 and relative potency of different formulations were determined. The ED50 of thimerosal-free formulations were reduced by more than 35% in both preparations. In addition, GMT of antibody titer, seroconversion and seroprotection indicated significantly higher immunogenicity for thimerosal free formulations for both aluminum phosphate and hydroxide adjuvants.

## Introduction

Thimerosal (sodium ethylmercurythio-salicylate) was developed by Eli Lilly in the 1930›s as an effective bacteriostatic and fungistatic preservative and has been widely used in multiple dose formulations such as vaccines including hepatitis B (1). Prior to its introduction data were available in several animal species and human providing evidence for its safety and effectiveness as a preservative. Since then, thimerosal has a long record of safe and effective use preventing bacterial and fungal contamination of vaccines. A vaccine containing 0.01% thimerosal contains 50 µg of thimerosal or approximately 25 µg of mercury per 0.5 mL dose (2, 3). The problem with thimerosal is that it contains 49.6% mercury by weight which may cause neurotoxicity in humans, especially in fetuses, neonate and infants whose brains are still developing (4). Since the largest human exposure to mercury (µg/kg body weight) occurs in children under 18 months of age undergoing routine childhood immunization schedules, the use of mercury-containing preservatives in vaccines had declined markedly since 1999 based on recommendations by some regulatory and advisory bodies in Europe and North America (5, 6).As many vaccines given to children in developing countries still contain thimerosal, the removal of thimerosal from pediatric vaccines has become an important objective. In the present study, in order to evaluate the effects of thimerosal on immunogenicity of aluminum adjuvanted recombinant hepatitis B vaccine, thimerosal-free and thimerosal-containing formulations of the vaccine were compared in Balb/c mice. For this purpose, a thimerosal-free formulation of recombinant hepatitis B antigen that contains no preservative has been developed using aluminum hydroxide (Alhydrogel) and aluminum phosphate (Adju-Phos) as adjuvant. Then these two preparations have been compared with thimerosal-containing formulations. The immune response to thimerosal-free and thimerosal-containing aluminum adjuvanted hepatitis B vaccine were evaluated by comparing the geometric mean titer (GMT, mIU/mL), the rate of seroconversion, seroprotection, ED50 (ng) and relative potency in Bulb/C mice after 28 days of inter peritoneum (IP) injection. Pre-clinical analysis in Balb/c mice have demonstrated superior efficacy of thimerosal-free hepatitis B vaccine containing aluminum phosphate as an adjuvant. It should be noted that in the previous work, we have shown that aluminum phosphate shows more adjuvanticity than aluminum hydroxide in recombinant hepatitis B vaccine formulation (9). 

## Experimental

*HBs antigen*

The recombinant hepatitis B surface antigen used in this study was produced in *Pichia pastoris*, a histidine requiring strain, GS115 (his4) containing the gene for the *adw* subtype of HBsAg and was obtained from a local manufacturer (Darou Pakhsh Pharma. Co., Tehran, Iran). Purity and assay of the HBsAg was determined according to British pharmacopeia (8). Antigen content of bulk was assessed using ELISA technique (Hepanostika HBsAg Uniform ELISA kit, Biomerieux, Netherlands) and purity of HBsAg was determined by conducting reducing SDS-PAGE, Laemmli method using electrophoresis system (Mini-PROTEAN® 3 Cell, BIO-RAD, USA)(9). Total protein content of the bulk antigen was determined using bicinchoninic acid (BCA) method (Thermo scientific Pierce, USA). The content of residual host cell DNA in the bulk purified antigen was determined by semi-quantitative PCR technique using 5’-pd (T)12-18-3’(Amersham Biosciencces, USA) as a primer and 35 thermo cycles. The content of carbohydrates in bulk preparation was assessed based on Anthrone method using glucose (1 mg/mL Sigma, USA) as standard. Total lipids were determined by the colorimetric method using vanillin-ortho-phosphoric acid as reagent and cholesterol (Sigma, USA) as lipid standard.

*Vaccine formulations*

Aluminum phosphate (Adju-Phos®) and aluminum hydroxide (Alhydrogel ®) were purchased from Brenntag Biosector (Denmark). Concentrations of the adjuvants were calculated on the basis of their aluminum contents. Vaccine was formulated by mixing the HBsAg bulk in phosphate buffer (pH 7.4) containing dibasic sodium phosphate anhydrous (1.12 g/L) and monobasic sodium phosphate monohydrate (1.1 g/L) with aluminum hydroxide or aluminum phosphate in 0.9% NaCl without (as single dose vaccine) and with thimerosal (0.05 mg/mL, Sigma, USA, as multiple dose vaccine). The mixture was shaken on a reciprocal shaker (Kühner ISF-1-W, Switzerland)at 25°C and 140 rpm for 6 h and then at 4°C for 18 h. The final concentration of HBsAg and aluminum was 20 μg/mL and 500 ppm respectively. Dilutions of 1:512 (0.03906 μg/mL of antigen), 1:64 (0.3125 μg/mL of antigen) and 1:8 (2.5 μg/mL of antigen) of vaccine were prepared by addition of phosphate buffer, pH 7.4 containing 500 ppm of the relevant aluminum adjuvant. Engerix-B® (GSK, Belgium, Lot No: AHBVB127AG) hepatitis B vaccine which included aluminum hydroxide as adjuvant was used as a control vaccine. Formulations containing only aluminum adjuvants with and without thimerosal were served as negative control.

**Figure 1:**
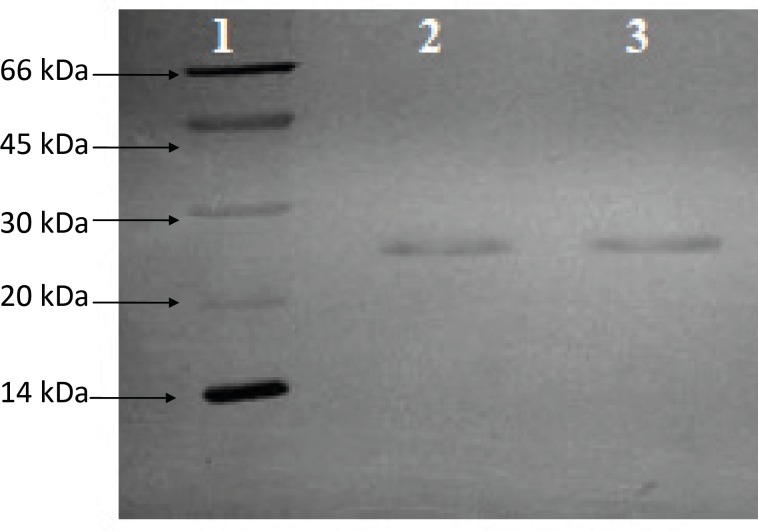
SDS-PAGE analysis of purified HBsAg. Thegel was stained with silver nitrate. Lane 2 and.3 purified bulk of HBsAg, Lane 1. Low molecular weight calibration kit (14.4-97KDa, GE Healthcare), Lane 3.Diluted (1:2) purified HBsAg bulk.

*Animals*

Female mice of Balb/c C3H strainsof 5-6 weeks old were obtained from Charles River Laboratories (Germany) and housed in Micro-Isolator™ at 25°C, 12 h day and night cycle with 50 ± 5% of relative humidity. Food (5 g) and water (6 mL) were served, for each mouse daily. All studies were performed in accordance with the procedures issued by the Institutional Animal Care and Use Committee. Each dilution of vaccine at volume of 1 mL was administered interperitoneally to 15 mice. To determine HBs antibody titer, the blood samples were collected from the heart of anaesthetized animal after 28 days. The serum of the blood samples were separated by centrifuging at 3000×g for 10 min and stored at -20°C.

*Determination of anti HBs titers*

Anti-HBs antibody was determined by ELISA technique using Diasorin (ETT-AB-AUK-3 anti-HBs antibody ELISA kit, Italy) according to the manufacture protocol. The seroprotection level was considered to be achieved when the antibody titer was at least 10 mIU/mL and antibody titer between 1mIU/mL to 10 mIU/mL was considered as seroconversion.

**Figure 2:**
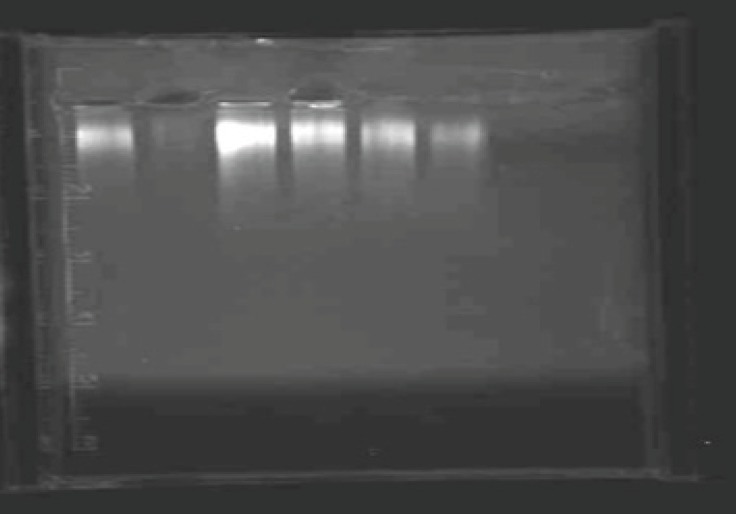
Determination of residual host cell DNA using semi-quantitative PCR on agarose gel electrophoresis. Lanes1, 3, 4, 5 and 6 are the positive controls containing 10, 100, 50, 25 and 5 pgs of the host cell derived DNA respectively and lane 2 is the sample and lane 7 is the negative control lacking either the host DNA or the sample.

*Statistics*

ED50 for each formulation was evaluated by SPSS VER.16 using Probit method while the relative potency of each formulation was assessed by quantal responses method (7). Geometric mean of anti-HBsAg titers (GMTs) were calculated by taking the anti-log of the mean of the log titer transformations. Antibody titers below the cut-off the assay were given an arbitrary value of half the cut-off for the purpose of GMT calculation. GMT and other data processing were taken with Microsoft Excel 2007.

## Results

*Specifications of bulk antigen*

The concentration of bulk antigen which was determined by the Hepanostika HBsAg ELISA kit was 42.57 μg/mL. Total protein content of the bulk antigen which was determined by BCA method was 37.34 μg/mL. The ratio of total antigen/total protein was 1.14 which has met acceptance value of 0.8-1.4. The result on the purity of HBsAg assessed with SDS-PAGE is depicted in Figure 1. Single band of 24kDa are shown in lanes 2 and 3 which correspond to monomer of HBsAg indicating high purity of the antigen which was used.

Determination of residual host cell DNA by semi-quantitative PCR showed less than 10 pg of DNA/dose of the vaccine (Figure 2). The lipid content of HBsAg was 0.33 mg per mg of protein while carbohydrate content was 162 μg per mg of protein which is indicative of acceptable quality of the used antigen (7).

**Figure 3:**
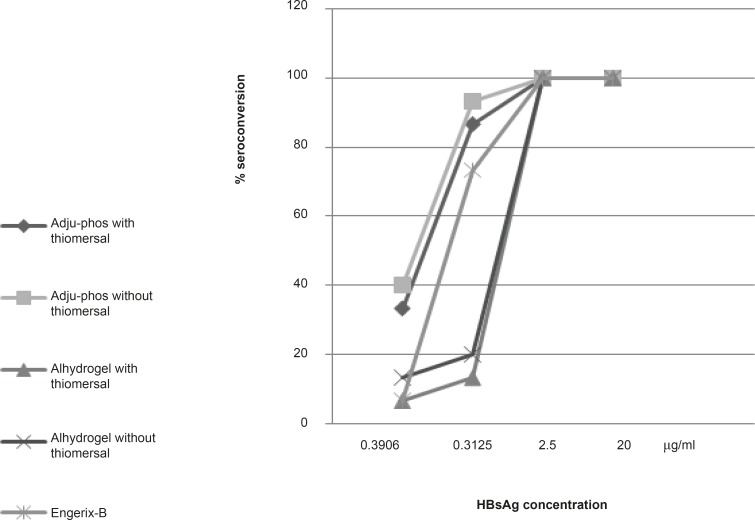
Seroconversion effect of different dilutions of hepatitis-B vaccines containing Adju-Phos (aluminum phosphate), Alhydrogel (Aluminum hydroxide) thimerosal-free or containing formulations and Engerix-B after 28 days of IP injection in Balb/c mice.

*Seroconversion of the formulated vaccines*

The results of 28 days seroconversion rates of various dilutions of hepatitis-B vaccine formulations containing aluminum phosphate or aluminum hydroxide (with and without thimerosal) and also those of the control vaccine is shown in Figure 3. All formulations showed 100% of seroconversion at concentrations of 2.5 μg/mL and higher while at lower HBsAg concentrations (0.3125 μg/mL, 0.039 μg/mL), thimerosal-free formulations showed higher seroconversion rate in both aluminum hydroxide (20%, 13.33%)and phosphate (93.33%, 40%) adjuvanted vaccines compared to thimerosal-containing aluminum hydroxide (13.33%, 6.66%) and phosphate (86.66%, 33.33%) based formulations.

*ED50*

**Figure 4:**
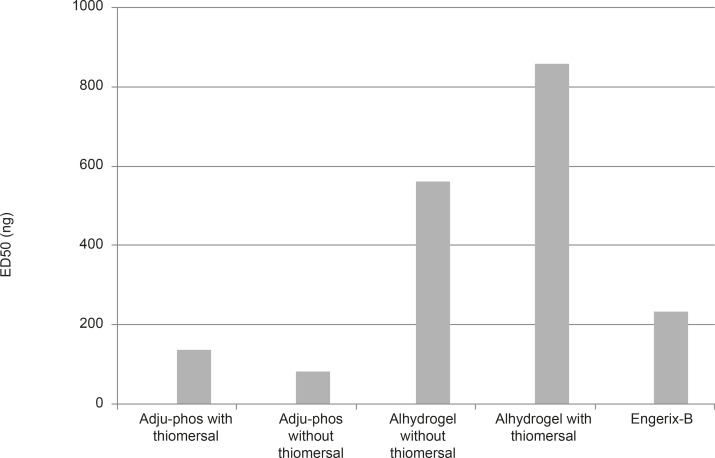
ED50 of different formulations containing Adju-Phos (aluminum phosphate) or Alhydrogel (Aluminumhydroxide) thimerosal-free or containing formulation and Engerix-B after 28 days of IP injection in Balb/c mice (p < 0.05).

ED50 (The dose which could induce seroconversion in 50% of the vaccinated population) for individual formulations were calculated with statistical software package SPSSVER.16 using Probit method with 95% confidence limit and the results are shown in Figure 4. According to results obtained, the ED50 of the thimerosal-free aluminum phosphate and hydroxide formulated vaccine was considerably lower than thimerosal containing vaccine which indicates to better immunogenicity (by more than 150%).

*Seroprotection of the formulated vaccines*

**Figure 5:**
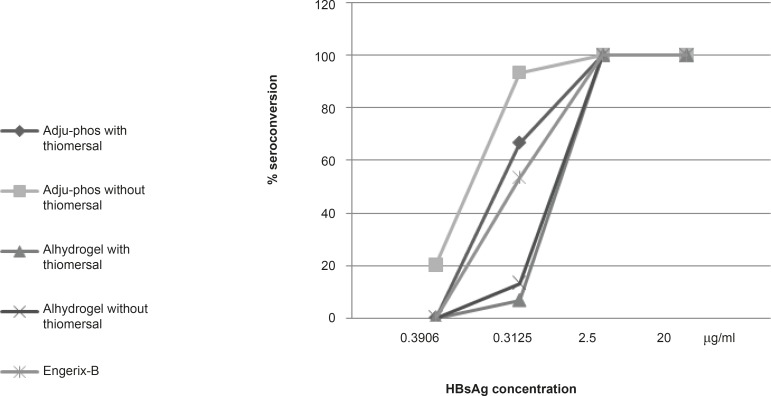
Seroprotection effect of different dilutions of hepatitis-B vaccines containing dju-Phos (aluminum phosphate), Alhydrogel (Aluminum hydroxide) thimerosal-free or containing formulations and Engerix-B after 28 days of IP injection in Balb/c mice

Seroprotection rates of various dilutions of hepatitis-B vaccine formulations containing aluminum phosphate or aluminum hydroxide (with and without thimerosal) and also those of the control vaccine are depicted in Figure 5. All formulations showed 100% of seroprotection at concentrations of 2.5 μg/mL and higher while at lower HBsAg concentrations (0.3125 μg/mL,0.039 μg/mL), thimerosal-free formulations showed higher seroprotection rate (93%, 20% for aluminum phosphate) and (13.33% and 0% for aluminum hydroxide) compared to thimerosal-containing based formulations (66.66%, 0% in aluminum phosphate and 6.66%, 0% in aluminum hydroxide formulation).

*Geometric mean of antibody titers*

GMT titers of different formulations of hepatitis B vaccine are depicted in Table 1. Using a concentration of 20 μg/mL antigen the titer of GMT obtained with thimerosal-free aluminum phosphate formulation (4217.64 mIU/mL) was higher compared to thimerosal containing based formulation (2573.94 mIU/mL) and also that of control vaccine (810.25 mIU/mL). These amounts at lower concentration of antigen (0.039 μg/mL) were respectively 165.52 and 4.12 (mIU/mL). Also, this pattern has been repeated in aluminum hydroxide formulation (with and without thimerosal). Accordingly, at concentration of 20 μg/mL the GMT of antibody titer in thimerosal-free and containing vaccine was respectively 956.4 and 689.24 mIU/mL.

*Relative potency*

**Figure 6:**
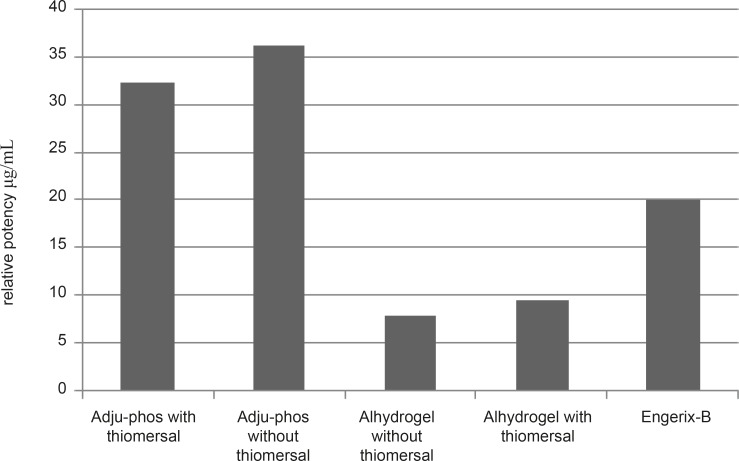
Relative potency different formulations containing Adju-Phos(with and without thimerosal) or Alhydrogel (with and without thimerosal) and Engerix-B after 28 days of IP injectionin Balb/c mice.

According to the seroconversion rates the relative potencies of the individual formulations were estimated using the quantal responses method (7). Engerix–B vaccine was used as the reference vaccine. Relative potency of different formulation of hepatitis B vaccines are shown in Figure 6. The relative potency of thimerosal-free formulations of hepatitis B vaccines adjuvanted with aluminum phosphate and aluminum hydroxide was considerably higher than those of thimerosal-containing based formulation.

## Discussion

Thimerosal is an organomercury compound used to prevent microbial growth during the manufacturing process or as a preservative in «multi-dose» vaccines (1).Toxicity of mercury which formed 49.6% constituent of thimerosal has been linked to many different diseases, including autism and learning disabilities (10, 11). Following a mandated review of mercury-containing food and drugs, the Centers for Disease Control and Prevention (CDC) and some other regulatory bodies asked vaccine manufacturer to eliminate thimerosal from vaccines as quickly as possible as a precautionary measure to minimize exposure of infants and children to mercury, and it was rapidly phased out of most developed countries vaccine products(1, 2, 12).With the exception of influenza vaccines, all vaccines manufactured since 2001 that are routinely recommended in the in Europe and North America for children 6 years of age and under are presented in single-dose formulations and do not contain thimerosal as a preservative. As with pediatric vaccines, exposure to thimerosal in vaccines for adolescents and adults has also been reduced or eliminated because of growing concerns. Thus, the use of thimerosal preservative in FDA-licensed vaccines has significantly declined over the last decade (1, 2, 13). In spite of this, many vaccines given to children in developing countries still contain thimerosal.

Regarding to considerable concern with neurotoxicity of mercury and its potential risk of neurodevelopmental disorders, in this study the single dose thimerosal-free hepatitis B vaccine were developed and preclinical study of these formulations were conducted. Accordingly, the effects of thimerosal on immunogenicity of single dose aluminum adjuvanted (Aluminum phosphate, Aluminum hydroxide) recombinant hepatitis-B vaccine were evaluated in Balb/c mice in comparison with multiple dose formulation containing thimerosal. Based on the obtained results, in spite of thimerosal elimination from formulation, Adju-phos adjuvanted hepatitis B vaccine has shown approximately 11% increase in relative potency, 163% in GMT and 41% reduction in ED50 at 20 µg dose of vaccine. Also, similar results have been observed in Alhydrogel adjuvanted vaccine without thimerosal in comparison with thimerosal containing formulation. The relative potency and GMT of thimerosal-free Alhydrogel adjuvanted vaccine have shown respectively 18.5% and 138% increase at the 20 µg dose of vaccine while the ED50 reduced about 35%. The increased immunogenicity of thimerosal-free formulation may be due to less ligand exchange between phosphate group of the antigen and aluminum adjuvant leading to less stable binding of the adjuvant to antigen. Furthermore, altering in antigen presenting mechanism to immunocompetent cells such as interleukins and tumor necrosis factor (TNF) may be occurred in presence of thimerosal at injection site. Another explanation for reducing immune response and cytotoxicity of thimerosal against immune cells is attributed to the high affinity of mercury compound to the thiol (SH) group of cysteine in structure of HBsAg. Also, a recent in vitro study of thimerosal cytotoxicity using immortalized Jurkat T-cell demonstrated an increase in reactive oxygen species and a decrease in intracellular glutathione with increasing concentration of thimerosal. Thimerosal, but not thiosalacylic acid (the non-mercury component of thimerosal), induced apoptotic cell death in T cells in a concentration-dependent manner as evidenced by mitochondrial release of cytochrome c, apoptosis activating factor, and activation of caspases 9 and 3. Exogenous glutathione inhibited activation of these caspases and prevented cell death (14).

**Table 1:** Geometric mean antibody titers (GMT) of different formulations containing Adju-Phos (Thimerosal-free or containing formulation) or Alhydrogel(Thimerosal-free or containing formulation) and Engerix-B after 28 days of IP injection in Balb/c mice.

**Vaccine**	**Dose (μg/mL) GMT**	**GMT (mIU/mL)**	**Standard Deviation (mIU/mL)**
Adju-phos with thimerosal	20	2573.94	1652.34
	2.5	945.41	387.54
	0.3125	165.52	62.48
	0.03906	4.12	2.63
Adju-phos without thimerosal	20	4217.64	2364.7
	2.5	998.59	325.54
	0.3125	277.16	97.63
	0.03906	7.13	3.42
Alhydrogel with thimerosal	20	689.24	352.56
	2.5	320.62	181.76
	0.3125	1.38	2.44
	0.03906	1.26	2.88
Alhydrogel without thimerosal	20	956.4	380.43
	2.5	595.2	193.5
	0.3125	4.62	4.22
	0.03906	2.03	1.23
Engerix-B	20	810.25	453.22
	2.5	524.49	312.45
	0.3125	43.22	28.35
	0.03906	1.21	1.8

Based on the result of the present study it could be suggested that elimination of thimerosal in formulation of hepatitis-B vaccine especially in routinely recommended childhood not only reduce the concerns on side-effect of the thimerosal but also lead to superior immune response. Also, the risk assessment of thimerosal use in pediatric vaccines as a highly debatable topic still remains a subject of ongoing scientific investigation.
